# Donepezil Impairs Memory in Healthy Older Subjects: Behavioural, EEG and Simultaneous EEG/fMRI Biomarkers

**DOI:** 10.1371/journal.pone.0024126

**Published:** 2011-09-08

**Authors:** Joshua H. Balsters, Redmond G. O'Connell, Mary P. Martin, Alessandra Galli, Sarah M. Cassidy, Sophia M. Kilcullen, Sonja Delmonte, Sabina Brennan, Jim F. Meaney, Andrew J. Fagan, Arun L. W. Bokde, Neil Upton, Robert Lai, Marc Laruelle, Brian Lawlor, Ian H. Robertson

**Affiliations:** 1 Trinity College Institute of Neuroscience and School of Psychology, Trinity College Dublin, Dublin, Ireland; 2 Trinity College Institute of Neuroscience and School of Medicine, Trinity College Dublin, Dublin, Ireland; 3 Mercer's Institute for Research on Ageing, St. James's Hospital, Dublin, Ireland; 4 Centre for Advanced Medical Imaging (CAMI), St. James's Hospital, Trinity College Dublin, Dublin, Ireland; 5 Neurosciences Centre of Excellence for Drug Discovery, GlaxoSmithKline, Harlow, United Kingdom; 6 Neurosciences Discovery Medicine, GlaxoSmithKline, Harlow, United Kingdom; City of Hope, United States of America

## Abstract

Rising life expectancies coupled with an increasing awareness of age-related cognitive decline have led to the unwarranted use of psychopharmaceuticals, including acetylcholinesterase inhibitors (AChEIs), by significant numbers of healthy older individuals. This trend has developed despite very limited data regarding the effectiveness of such drugs on non-clinical groups and recent work indicates that AChEIs can have negative cognitive effects in healthy populations. For the first time, we use a combination of EEG and simultaneous EEG/fMRI to examine the effects of a commonly prescribed AChEI (donepezil) on cognition in healthy older participants. The short- and long-term impact of donepezil was assessed using two double-blind, placebo-controlled trials. In both cases, we utilised cognitive (paired associates learning (CPAL)) and electrophysiological measures (resting EEG power) that have demonstrated high-sensitivity to age-related cognitive decline. Experiment 1 tested the effects of 5 mg/per day dosage on cognitive and EEG markers at 6-hour, 2-week and 4-week follow-ups. In experiment 2, the same markers were further scrutinised using simultaneous EEG/fMRI after a single 5 mg dose. Experiment 1 found significant negative effects of donepezil on CPAL and resting Alpha and Beta band power. Experiment 2 replicated these results and found additional drug-related increases in the Delta band. EEG/fMRI analyses revealed that these oscillatory differences were associated with activity differences in the left hippocampus (Delta), right frontal-parietal network (Alpha), and default-mode network (Beta). We demonstrate the utility of simple cognitive and EEG measures in evaluating drug responses after acute and chronic donepezil administration. The presentation of previously established markers of age-related cognitive decline indicates that AChEIs can impair cognitive function in healthy older individuals. To our knowledge this is the first study to identify the precise neuroanatomical origins of EEG drug markers using simultaneous EEG/fMRI. The results of this study may be useful for evaluating novel drugs for cognitive enhancement.

## Introduction

The incidence of cognitive impairment rises with age, with 5% of 71–79 year olds showing dementia, rising to 37.4% of 90 year olds and above [1]. The proportion of people over 70 is projected to rise dramatically in the coming years. In the United Kingdom, for instance, the life expectancy at birth for those born in 2009 is projected to be around 90 years (88.7 years for males and 92.3 years for females) [2]. Currently, the life expectancy for those aged 65 is projected to be around 85 years (86.1 years for males and 88.8 years for females) [2]. This demographic change is likely to be accompanied by a mushrooming of the number of people with dementia and age-related cognitive deficits. The health, social and economic burden that this will present to society will be formidable unless methods can be identified to delay cognitive decline among people in their 60′s, 70′s and even 80′s.

Perhaps reflecting a growing awareness of the impact of age-related cognitive decline amongst the general public, a recent poll in the journal *Nature* [3] found that a large number of elderly people (55–65 year olds) are seeking psychopharmaceuticals as a means of improving their cognitive function with acetylcholinesterase inhibitors (AChEIs) such as donepezil, rivastigmine and galantamine being the most commonly prescribed [4], [5]. Donepezil is the most prescribed pharmaceutical for the treatment of Alzheimer's Disease (AD) and whilst it has proven effective in treating mild to moderate AD, there are limited data on either the cognitive or neural impact when administered to healthy older individuals. A recent review of AChEI administration in healthy older participants [6] found only 13 relevant studies of which 12 were on the effects of donepezil. The findings of these studies were inconsistent, but generally suggested that AChEI administration had either no effect or negative effects on healthy individuals. When positive effects of donepezil were found it was either in the oldest populations (over 70) [7] or under strenuous circumstances such as sleep deprivation [8], [9]. To date only three AChEI studies have provided neuroimaging data on healthy young individuals [8], [9], [10], none have yet been conducted on healthy older individuals.

One possible reason there are no imaging studies of AChEI's in healthy older individuals is the difficulty in interpreting pharmacological functional Magnetic Resonance Imaging (fMRI) results. In most fMRI studies it is likely that drugs not only modulate neural activity but also modulate the intervening stages between neural activity and the BOLD response such as synaptic/metabolic signalling or vascular responsiveness which can lead to false positives or false negatives [11], [12], [13]. Whilst these confounds can be addressed to some degree (good control conditions, additional physiological recordings, and perfusion imaging) the combination of fMRI with a more direct measure of neural activity, such as electroencephalography (EEG), is highly advantageous. EEG is a more direct measure of neural activity in comparison to fMRI and has the additional advantage of unparalleled temporal resolution. FMRI offers a significant increase in spatial resolution compared to EEG, including the ability to interrogate neural changes in subcortical structures like the basal ganglia and the thalamus. Fusing these two complementary methods has the potential to greatly improve the quality of neuroimaging research in more challenging situations such as studies of drugs and disease [11]. Whilst this multi-modal approach is still in its infancy, the majority of technical challenges (gradient and balistocardiogram removal) have been successfully addressed [14], [15], [16], [17]. To our knowledge this is the first simultaneous EEG/fMRI study investigating the effects of a pharmacological agent.

Here, we present two double-blind, randomised, placebo-controlled trials investigating the effects of donepezil on healthy older subjects. In both cases, we utilised cognitive (continuous paired associates learning (CPAL)) and electrophysiological measures (resting EEG power) that have previously demonstrated high-sensitivity to age-related cognitive decline [18], [19]. Experiment 1 tested the effects of 5 mg/per day donepezil dosage on cognitive and EEG markers at 6-hours after the first dose, 2-weeks and 4-weeks of treatment. In experiment 2, the same markers were further scrutinised using simultaneous EEG/fMRI 6-hours after a single 5 mg dose. As experiment 2 was a single dose study, a cross-over design was employed for further sensitivity. An extensive EEG literature has demonstrated that the progression of age-related cognitive decline is reliably traced by changes in spectral profile, characterised by increasing delta-band power and decreasing alpha band power, and this same pattern is exaggerated in patients with dementia compared to healthy controls [18], [19], [20]. Given the inconsistent results reported by previous studies of donepezil treatment in healthy elderly participants we proposed a two-tailed hypothesis whereby positive donepezil outcomes would be accompanied by decreases in Delta power, increases in posterior Alpha power, and improved memory performance, that is, a reversal of the negative cognitive and EEG trends reported in previous studies of cognitive decline. Negative donepezil outcomes would be accompanied by the opposite trends, increased Delta power, diminished posterior Alpha power and poorer memory performance.

## Results

### Experiment 1: Double blind, parallel group, placebo-controlled trial of donepezil administration (5 mg per day) over 4 weeks using cognitive and EEG assessments

#### Continuous Paired Associates Learning (CPAL)

**Figure 1:**
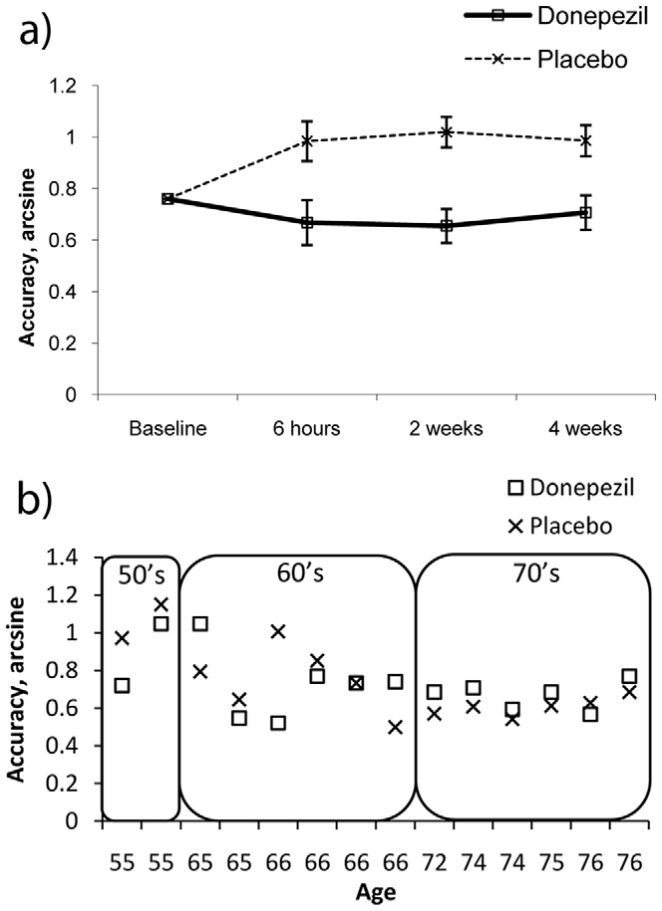
CPAL performance: In both graphs a larger score equates to better performance. a) Age, baseline performance, IQ and MMSE adjusted CPAL scores from experiment 1. Significant negative effects occur as early as 6 hours post donepezil. b) Individual subjects arcsine transformed CPAL scores from experiment 2. The x axis shows the individual subject's age (boxes indicate decade boundaries; 50′s, 60′s, 70′s). This highlights the treatment by age interaction (negative effect of donepezil for participants in their 50′s, positive for participants in their 70′s). Values in figure 1b have not been adjusted for covariates i.e. baseline score or session effects.

A main effect of treatment was observed for the CPAL, F(1,14)  = 11.79, p<0.01, η^2^ = 0.46. As illustrated in Figure 1a, the placebo group showed a strong practice effect over 4 weeks (29% improvement) but the performance of the donepezil group deteriorated slightly (7% decrement). Further analysis indicated that these effects were already apparent at the 6-hour follow-up, F(1,14) = 6.1, p<0.05, η^2^ = 0.3. Untransformed CPAL values are presented in Table S1. Two additional subtests of the Cogstate [21] research battery were included (one-back working memory test and a simple reaction time test) but these failed to show any significant drug effects.

#### Resting EEG

**Figure 2:**
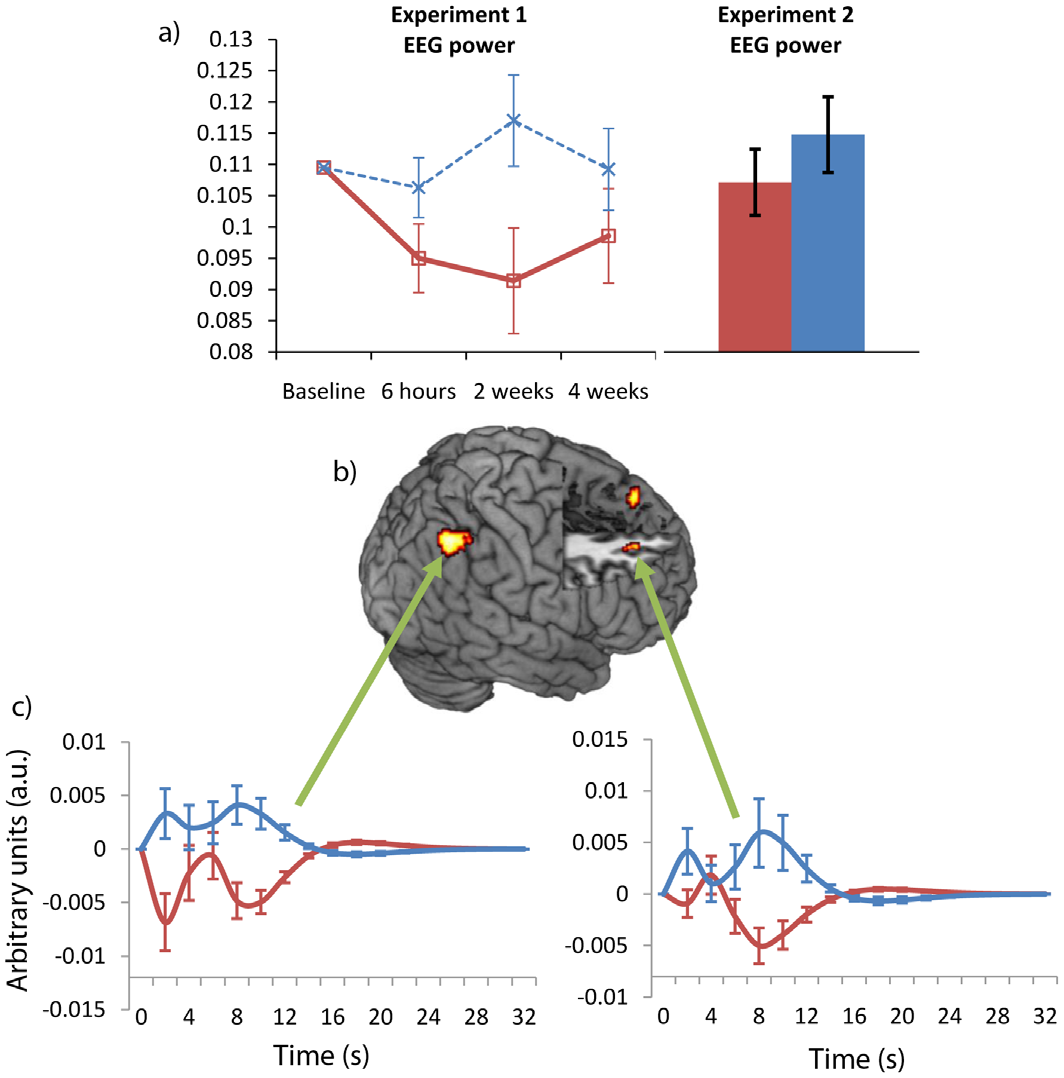
Tonic Alpha 2 (11-14 Hz) Drug effects: a) Resting EEG power from experiment 1 and experiment 2 both show significant decreases in relative Alpha power on donepezil. b) Changes in Alpha 2 EEG power were mapped to right frontal-parietal regions. c) Graphs show the Alpha response functions from highlighted regions. In all graphs red corresponds to donepezil, blue to placebo.

Statistical analysis revealed a significant Treatment by Region interaction for relative Alpha 2 power in the eyes closed condition, F(2,24) = 3.9, p<0.05, η^2^ = 0.25 and a non-significant trend toward a main effect of Treatment F(1,12) = 3.5, p = 0.08, η^2^ = 0.2. Post-hoc contrasts indicated that the interaction was driven by a significant main effect of Treatment at parietal electrodes, F(1,14) = 10.1, p<0.01, η^2^ = 0.42, reflecting a decrease in Alpha power in the donepezil group across the one-month follow-up period (see Figure 2a). Further analysis showed a significant positive correlation with CPAL score and relative alpha power at the 2week session (r = 0.5, p<0.05) whereby better performance on the CPAL was associated with larger relative alpha power. The same positive trend was also apparent at 6 hr (r = 0.3, p = 0.18) and 4 week (r = 0.1, p = 0.6) follow-ups but did not reach significance.

In the eyes open resting condition we observed a significant main effect of Treatment for relative Beta power F(1,11)  = 3.9, p<0.05, η^2^ = 0.35 driven by an increase in Beta power across frontal, central and parietal electrodes in the donepezil group versus placebo.

### Experiment 2: Double blind, crossover design, placebo-controlled trial of single dose (5 mg) donepezil using cognitive and simultaneous EEG/fMRI assessment

Pharmacodynamic markers of donepezil which showed significant effects from experiment 1 were reassessed in experiment 2 using different subjects.

#### Continuous Paired Associates Learning (CPAL)

As in experiment 1, participants performed significantly worse on the CPAL 6 hrs after receiving donepezil compared to placebo F(1,10)  = 6.14, p<0.05, η^2^ = 0.38. Experiment 2 also showed a significant treatment by age interaction, F(1,10)  = 5.55, p<0.05, η^2^ = 0.36, illustrating that the negative effects of donepezil decreased with age (see figure 1b). Untransformed CPAL values are presented in Table S1.

#### Resting EEG

The EEG power trends (increasing delta, decreasing alpha) of experiment 1 were replicated in experiment 2 (plots for significant results shown in figure S1). Significant drug-related increases in Delta EEG power, F(1,11)  = 9.68, p<0.05, η^2^ = 0.47, and decreases in Alpha 1 EEG power, F(1,11)  = 10.38, p<0.05, η^2^ = 0.49, were observed during the eyes closed conditions. Significant interactions included a drug by session interaction in Alpha 1, F(1,11)  = 12.53, p<0.05, η^2^ = 0.53, showing that the drug effect was eliminated if donepezil was administered first, and a drug by age interaction for Delta, F(1,11)  = 6.99, p<0.05, η^2^ = 0.39, that reflected a less negative drug effect for older participants.

A significant drug by region interaction was observed during the eyes open condition for Alpha 2 EEG power, F(5,55)  = 3.88, p<0.05, η^2^ = 0.26, reflecting decreased activity on donepezil over parietal (T(1,13)  = −2.56, p<0.05), occipital (T(1,13)  = −2.363, p<0.05)), and right temporal regions (T(1,13)  = −3.63, p<0.005). There was also a significant drug by region by session interaction showing the drug effect (decreased Alpha 2 EEG power on donepezil) was eliminated from the left temporal region if donepezil was taken first, F(5,55)  = 2.67, p<0.05, η^2^ = 0.2. Finally there was a drug by region by age interaction showing that donepezil elicited greater Alpha2 EEG power in the right temporal region in participants under 70 and greater Alpha 2 EEG power in the central region in participants over 70, F(5,55)  = 4.67, p<0.05, η^2^ = 0.3. All these EEG drug effects for both eyes open and eyes closed conditions are illustrated in figure S1.

It is also worth noting a near significant drug effect on Beta EEG power, whereby donepezil administration lead to greater relative Beta power compared to placebo (p = 0.06). Participants also showed near significant drug by age interactions in both eyes open (p = 0.051, decreased Beta activity on donepezil for participants under 60) and eyes closed conditions (p = 0.054, increased Beta activity on donepezil for participants over 70).

#### Resting EEG/fMRI

Experiment 2 used a different resting task to experiment 1 where the participant alternated between eyes-open and eyes-closed conditions every 30 s. This allowed us to model two types of EEG oscillatory markers within each frequency band; EEG reactivity (EEG power that changed between eyes-open and eyes-closed conditions) and tonic EEG power (EEG power unaffected by the transition between eyes-open and eyes-closed conditions). This design gave us the opportunity to further scrutinise EEG oscillatory markers, particularly Alpha [22] which is a consistent marker of cognitive function, age-related cognitive decline, and AD [23], [24]. Further details of drug effects on tonic EEG power and EEG reactivity are included in the supplemental results (Results S1). The simultaneous EEG/fMRI results are reported in Table 1.

**Table 1:** Table of activations for drug-related differences in EEG/fMRI.

	Cluster	F	Co-ordinate				Cytoarchitectonic BA	BOLD
Alpha 2 Reactivity	Size		(x y z)				(Probability if available)	Response
Right Superior Medial Gyrus	13	7.35	12	38	52		Area 9	Placebo +ve
Right Anterior Cingulate	12	7.41	8	36	12		Area 24	Placebo +ve
Left Anterior Cingulate	14	6.98	−2	26	24		Area 24	Drug +ve
Right Rolandic Operculum	16	6.67	52	−22	16		OP1 (84%)	Drug +ve
**Delta Tonic**								
Right Superior Medial Gyrus	39	9.94	10	58	24		Area 10	Drug +ve
Right Middle Cingulate	49	7.69	10	0	32		Area 24	Drug +ve
Left Superior temporal Gyrus	32	7.99	−54	−14	−4		TE 1.2 (10%)	Drug +ve
Left Parahippocampal Gyrus	13	7.02	−18	−24	−20		Hipp (sub)(90%)	Drug +ve
**Alpha 2 Tonic**								
Left Superior Medial Gyrus	48	7.45	−12	56	0		Area 10	Drug –ve
Left Superior Medial Gyrus	20	7.74	−8	40	46		Area 9	Drug –ve
Right Superior Frontal Gyrus	15	7.73	18	40	28		Area 9	Drug –ve
Left Middle Frontal Gyrus	27	8.26	−36	18	40		Area 44 (10%)	Drug –ve
Right Inferior Parietal Lobule	95	9.04	40	−56	46		hIP1 (30%), hIP3 (30%), IPC (Pga)(20%)	Drug –ve
**Beta Tonic**								
Right Superior Medial Gyrus	70	8.3	10	52		2	Area 10	Placebo –ve
Right Anterior Cingulate	14	6.97	2	50		14	Area 32	Drug +ve
Right Superior Medial Gyrus	34	8.31	4	38		52	Area 6 (10%)	Drug +ve
Right Cerebellum	17	7.23	38	−56		−30	Crus I (71%), Lobule HVI (29%)	Drug –ve
Right Precuneus	18	7.31	6	−62		34	SPL(7A)(10%)	Placebo –ve
Right Angular Gyrus	24	7.34	46	−70		32	IPC(PGp)(90%)	Drug D –ve

Cluster size indicates the number voxels active in each cluster. The BOLD response column indicates the direction of the BOLD effect: ‘Drug’/’Placebo’ refers to whether the difference was driven by a change in activity in drug or placebo, ‘+ve’/‘-ve’ refers whether the change in activity was driven by positive or negative BOLD.

Both tonic Alpha 2 and Alpha 2 reactivity showed significant, but opposite, treatment effects in our combined EEG/fMRI analysis. Decreases in Alpha 2 reactivity on donepezil corresponded to increased BOLD activity on donepezil in the left anterior cingulate (area 24) and the right rolandic operculum (OP1 (84%)) and decreased BOLD activity in the right superior medial gyrus (area 24) and right anterior cingulate (area 24). Decreases in tonic Alpha 2 power on donepezil corresponded to decreased BOLD activity on donepezil in the left superior medial gyrus (areas 9 and 10), left middle frontal gyrus (area 44 (10%)), right superior frontal gyrus (area 9), and the right inferior parietal lobule (hIP1 (30%); hIP3(30%)). The right frontal-parietal network is highlighted in figure 2 given it is a previously established marker of age-related cognitive decline [25], [26].

**Figure 3:**
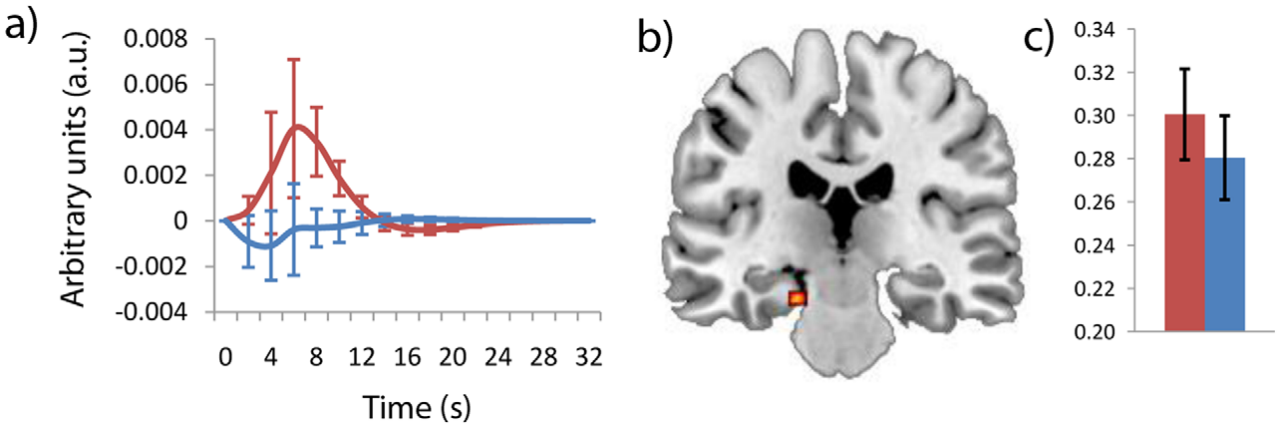
Hippocampal Delta (1.5-3 Hz) drug effects: a) Delta response function taken from the left hippocampus (subiculum 90%). b) Hippopcampal activation driven by differences in Delta EEG power. c) Relative Delta band EEG power differences from experiment 2. In all graphs red corresponds to donepezil, blue to placebo.

Treatment effects were also present in tonic Delta EEG/fMRI (increase in Delta EEG power on donepezil) showing increased BOLD activity on donepezil in the superior medial gyrus (area 10), middle cingulate (area 24), left superior temporal gyrus (T.E. 1.2 (10%)) and the left hippocampus (subiculum (90%)). Figure 3 shows the hippocampal activation along with its haemodynamic response function and relative EEG power differences. Given that both the hippocampus and the CPAL are strong predictors of cognitive decline and dementia a linear regression was run with CPAL score as a dependent variable and independent variables of age, session, and hippocampal beta values (taken from the Delta EEG/fMRI regressors). The results indicated a near significant relationship between hippocampal activity and CPAL performance (r = −0.441, p = 0.057 for drug and r = −0.445, p = 0.055 for placebo). In both cases reduced hippocampal activity was correlated with better CPAL performance.

**Figure 4:**
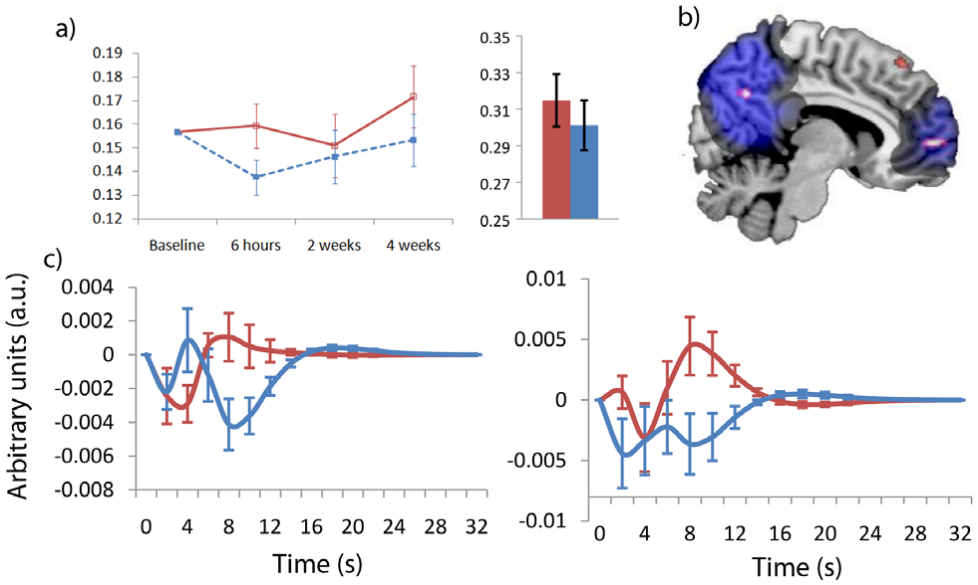
Beta (14-30 Hz) default mode drug effects: a) Resting EEG power from experiment 1 and experiment 2 both show significant increases in relative Beta power on donepezil. b) Beta EEG/fMRI drug differences overlayed on a reference image of the default mode network (blue). c) Graphs show Beta response functions to posterior (left graph) and anterior (right graph) activation clusters. In all graphs red corresponds to donepezil, blue to placebo.

Finally, increased tonic Beta EEG power on donepezil was mapped to decreased BOLD activity on donepezil within medial territories (right superior medial gyrus (area 10), right anterior cingulate (area 32) and right precuneus (area 7A (10%))). These regions have been consistently found to be active during rest and labelled the default mode network [27]. Figure 4 shows these results overlayed on a reference image of the default mode network shown in blue. Donepezil induced significant decreases in activity in the right angular gyrus (PGp (90%)), right superior medial gyrus (BA 6 (10%)) and prefrontal projecting regions of the right cerebellar hemisphere (Crus I (71%)).

## Discussion

The cholinergic system is critical in supporting cognitive function, and memory processes in particular, and has been heavily implicated in AD [28], [29], [30]. As a result, many pharmacological therapies for AD have been targeted toward potentiating cholinergic function. Donepezil increases cholinergic tone by delaying the breakdown of acetylcholine (ACh) in the synaptic cleft and has proven efficacy in treating mild to moderate AD [31]. Recently there has been interest in the potential for these same drugs to strengthen cognitive function in normal ageing [3], [4], [5]. Our study provides a detailed exploration of the neurophysiological impact of AChEI administration in healthy older individuals and in so doing provides the first ever combined EEG/fMRI exploration of a pharmacological intervention. Our data show that two separate groups of healthy elderly participants exhibited consistently negative cognitive and neurophysiological responses to donepezil treatment including a disruption of resting EEG oscillations, reduced default mode activation, and worsening memory performance on the CPAL. Furthermore, all of these drug signals were detectable within six hours of the first 5 mg dose. Although steady-state equilibrium only occurs after 14 days of donepezil treatment [32] experiment 1 showed that the effects of donepezil on cognitive function were still present at 2-week and 4-week follow-ups.

The literature on the effects of donepezil administration to healthy elderly individuals has been inconsistent with individual studies reporting negative, positive, or no effects [6]. One reason for this lack of consistency may be that donepezil's effectiveness is dependent on specific factors including age and mental state. For example, of the studies on healthy subjects that have reported positive donepezil effects, two included participants who were in a state of sleep deprivation [8], [9] and another included older age elderly participants (70 s +) [7]. This tallies with the observations that AChEI treatment benefits correlate with disease severity in patients with AD [33]. Our results also highlight an age-interaction with older participants showing slight improvements under medication on both behavioural and neurophysiological markers. Taken together, these findings suggest that future studies are warranted that would seek to characterise normal elderly sub-groups who may benefit significantly from cholinergic treatment.

### Neural Markers of donepezil administration

Resting recordings acquired from EEG or fMRI are an increasingly common measure of functional connectivity and are often used as markers of disease or pharmacological intervention [34], [35]. A large number of ageing studies have consistently highlighted diminished activity within the Alpha band (8–13 Hz) accompanied by an increase in the power of slower Delta (1–4 Hz) and Theta (4–8 Hz) frequency ranges. This pattern appears to be exaggerated in patients with AD who exhibit a further increase in Delta and Theta power, and decreased Alpha power relative to age-matched controls [20], [36], [37]. These pathophysiological trends are significantly reduced amongst patients who respond to long-term AChEI treatment [38], [39], [40]. In both experiment 1 and experiment 2 we observed the opposite effect in healthy older subjects, such that acute (6 hr) and chronic (up to 4-week) administration of donepezil led to increased Delta EEG power and decreased Alpha EEG power. These effects were accompanied by significantly reduced memory performance.

#### Effects of Donepezil on Alpha and the Right Frontal-Parietal Network

Tonic activity in the Alpha range is thought to reflect the cognitive and attentional resources available to an individual [23], [24]. Babiloni et al., [38] showed using EEG source analysis that posterior Alpha activity was the best EEG predictor of a patient's response to AChEI treatment. In both experiment 1 and 2 we found that donepezil administration led to a decreases in posterior relative Alpha power. Alpha EEG power has also been consistently mapped to the right frontal-parietal regions [41], [42] and in this study we show that donepezil targeted this same network. Activation of the right frontal-parietal network (but not the left) has been shown to decrease linearly when comparing healthy elderly individuals to patients with Mild Cognitive Impairment (MCI) and when comparing patients with MCI to patients with AD [25]. Other studies have also found that right parietal activity is related to donepezil induced changes in cognitive performance. Chuah et al., [8], [9] showed that decreased activity within the right intraparietal sulcus (IPS) was linked to worse performance on a visual short term memory task whereas participants that responded positively to donepezil showed increased activity within the right IPS. Our combined EEG/fMRI results confirm that the right frontal-parietal Alpha network provides a useful index of the efficacy of cholinergic interventions.

#### Effects of Donepezil on Delta and the Hippocampus

As with Alpha, changes in Delta EEG power are consistently found to be markers of cognitive decline in ageing [18], [43] and AD [20]. Using simultaneous EEG/fMRI, Dang-Vu et al., [44] showed that Delta EEG power correlated with activity in both medial and lateral structures of the prefrontal cortex. Consistent with this work our results indicate that drug-related differences in tonic Delta EEG power correlated to activity changes in medial prefrontal regions along with differences in the left superior temporal gyrus, and the left hippocampus (subiculum). Hippocampal volume is a strong predictor of cognitive decline and of MCI conversion to AD [45], [46]. The paired associates learning task is also a strong predictor of MCI conversion [47] and performance on this task has been repeatedly linked to hippocampal integrity [48], [49]. Although the MRI literature on donepezil treatment is relatively small, a large number of clinical studies have found that extended donepezil treatment (from 10 weeks to 2 years) typically leads to increases in hippocampal volume or resistance to hippocampal atrophy [50], [51], [52], [53], [54]. Hippocampal volume can also be used to predict patient response to donepezil treatment [51]. To date however, no studies of donepezil have looked at hippocampal *activity* as a marker of cognitive decline using fMRI or PET. There was a near significant relationship between hippocampal activity and CPAL performance showing that reduced hippocampal activity was correlated with better CPAL performance (p = 0.057 for drug; p = 0.055 for placebo). We believe these results suggest that donepezil had a negative impact on CPAL performance due to disruption of hippocampal function. Through the use of simultaneous EEG/fMRI we found that this disruption in hippocampal function was correlated with changes in relative Delta EEG power.

#### Effects of Donepezil on Beta and the Default Mode Network

The default mode network (DMN) is a term used to describe a network of regions including medial prefrontal cortex (BA 10 and anterior cingulate cortex), posterior cingulate/retrosplenial cortex, and bilateral inferior parietal lobules that are consistently found to be active during periods of rest [27]. The DMN is also proving to be a powerful marker of cognitive decline and disease [27], [35]. Activity within the DMN decreases with poorer performance on working memory and attention tasks [55], [56], [57], [58] and decreases linearly during healthy ageing, MCI and AD [25], [55], [57]. In this study we find a drug-related increase in relative Beta power corresponds to a drug-related decrease in activity within regions of the DMN (figure 4). To date no other studies on donepezil have reported differences within the DMN. One explanation may be that this is the first time the effects of donepezil treatment have been studied at rest. This study also has the advantage of a crossover design so each subject acts as their own control. It is possible that the additional sensitivity gained from this approach brought more subtle drug effects to statistical significance. Finally, the addition of more accurate electrophysiological information may have helped to address the confounds of pharmacological fMRI and correct for false negatives. However, it is not surprising that we find drug-related changes in the DMN given that; 1) it is a robust marker of cognitive decline [55], [56], [57], [58], and 2) the hippocampus (which this study and others have shown to be modulated by donepezil) plays a key role in the modulation of the DMN [55], [58], [59]. Wang et al., [58] found reduced connectivity with the right hippocampus and the DMN in AD, however, this was paired with increased connectivity between the left hippocampus and the right lateral prefrontal cortex (BA 8/9) which was suggested to be a compensatory mechanism. In this study and others [50], [51], [52], [53], [54], donepezil treatment has been shown to impact on hippocampal volume and function. We therefore suggest that these drug-related changes in hippocampal function may have a knock-on effect leading to reduced default mode activity.

Given the simple utility of the DMN as a biomarker, it was one of the first brain states to be investigated using simultaneous EEG/fMRI. Unfortunately, the results are mixed and confusing as every EEG oscillatory band has been found to correlate with the DMN [42], [44], [60], [61], [62], [63], [64]. Experiment 2 found regions of the DMN modulated by Beta EEG power which has been the most consistent EEG oscillatory marker of DMN activity [42], [60], [61]. Laufs et al., [60] and De Munck et al., [65] showed that it is important to model multiple frequency bands as the DMN can be explained by using Alpha EEG power as an independent variable [62] but is better explained by Beta EEG power compared to Alpha EEG power. Our analysis approach included all EEG bands in a single model (except gamma) and we replicate the results of Laufs et al., [60] further suggesting that Beta EEG power is the best EEG oscillatory correlate of the DMN.

### Conclusions

As cognitive enhancement through pharmacology becomes increasingly popular it is important that pharmaceuticals are tested not only on patient groups but also on healthy individuals. This not only informs us about the efficacy of a given drug, but also generates novel markers for the development of pharmaceuticals. This study adds to the small number of behavioural studies suggesting that donepezil does not enhance cognition in healthy individuals. For the first time, additional neural markers of cholinergic modulation have been established using EEG and simultaneous EEG/fMRI. These include increased Delta EEG power, decreased Alpha EEG power, increased hippocampal activity, and decreased activity within the default mode network. Previous clinical studies of AChEIs have reported that the magnitude of patients' drug response is correlated with disease severity [33]. The review by Repenatis et al [6] also showed that studies reporting positive effects of donepezil were in much older populations (over 70) [7], or populations with reduced cognitive performance due to an external challenge such as sleep deprivation [8], [9]. Our results also show that the negative effects of donepezil were more apparent in younger participants (50′s), and that small benefits begin to appear in participants over 70 yrs (figure 1b). We suggest that AChEI administration needs to be more selective and tailored to each individual. The markers presented in this study can be quickly administered to determine whether a patient is responding negatively to donepezil after as little as 6 hrs of a single 5 mg dose. These markers will also help the development of novel pharmaceutical interventions.

## Materials and Methods

### Screening Criteria

Prior to inclusion in the study all participants underwent a detailed physical, neurological and psychiatric examination, including laboratory blood tests, urine analysis and electrocardiogram. Participants were excluded for any contraindications to taking donepezil as specified in the summary of product characteristics. Further exclusion criteria included active medical or psychiatric illness, history of psychiatric or neurological illness or unexplained loss of consciousness, history of head injury, abnormal electrocardiogram, currently taking psychotropic medication, history or presence of drug use or dependency, history or presence of alcohol dependency, score above 8 on the depression or anxiety axes of the Hospital Anxiety and Depression Scale (HADS) [66], Mini-mental state exam (MMSE) [67] scores below 27, and exhibiting below normal age-adjusted memory performance at neuropsychological screening (see Procedures). For Experiment 2 there were additional standard MRI exclusion criteria including pacemakers, shunts, stents, artificial hip etc. All participants had normal or corrected to normal vision and had English as their first language. Participants were reimbursed for any travel expenses incurred during participation in the study.

Participants who met these criteria were randomly assigned to a drug or placebo group by a member of the pharmacy team who had no involvement with the study and no contact with any of the investigators. Experiment 2 was a crossover design so participants received both 5 mg donepezil and placebo at different stages of the study. The order of administration was randomised by the pharmacy team. All participants gave written informed consent and all procedures were approved by the Irish Medical Board and the ethical review boards of St James Hospital and the School of Psychology, Trinity College Dublin.

### Experiment 1: Double blind, placebo-controlled trial of donepezil administration (5 mg per day) over 4 weeks using cognitive and EEG assessment

#### Participants

A total of 24 healthy older participants were recruited to the study from the Dublin area. Four participants experienced adverse effects (severe nausea, diarrhoea, vomiting) and withdrew from the study before completing 2-weeks of treatment. A final sample of twenty participants (14 female) aged 59 to 77 (mean 67.5, SD = 5) completed the study.

#### Screening

This study was a four-week double-blind comparison of donepezil versus placebo across four separate testing sessions (24 hours pre-drug, 6 hours post-drug, 2 weeks post-drug, 4 weeks post-drug). All participants completed a medical and neuropsychological screening within ten days before the first baseline testing session. The neuropsychological screen consisted of the MMSE, the National Adult Reading Test (NART, estimate of intelligence) [68], a subjective memory self-rating scale, the HADS, the Stroop test, animal fluency and four subtests of the Wechsler Memory Test (WMS) [69]: Logical Memory, Visual Reproduction, Face Recognition and Digit Span. The delayed recall scores from each of the Logical Memory, Visual Reproduction and Face Recognition were used to exclude participants with potential early dementia (defined as a z-score greater than 1.5 standard deviations below the norm).

On completion of screening, a randomisation procedure was used by the hospital pharmacy staff to allocate participants to either the donepezil or placebo group. The randomisation was stratified to ensure a balanced male-female ratio in each group. Participants received either 5 mg of donepezil or placebo once a day for four weeks. The drug and placebo capsules were over-encapsulated in order to make them indistinguishable. Administration of the first dose took place in the hospital. Compliance was assessed at each of the post-drug testing sessions by checking the pill count of dispensed medication. Subjects, test administrators and all medical staff were blinded to the treatment subjects received. On completion of the study a full medical screening was repeated.

### EEG and Cognitive Testing

Each testing session was identical and consisted of a number of EEG-tailored cognitive tests and three subtests from the Cogstate Research battery [21]. Each session lasted approximately 90 minutes including frequent rest breaks. The EEG tests included a three minute eyes-closed resting recording, a three minute eyes-open resting recording, a two-stimulus auditory oddball task in which participants were presented with a series of tones and were required to make a mouse button press when they heard a low-probability (0.2) target tone (‘passive oddball’), a two-stimulus auditory oddball task in which participants were required to make a mouse button press following all tones except for the low-probability target tones (‘inhibitory oddball’). The Cogstate subtests that were administered were the continuous paired associates learning task (CPAL), the one-back task and the detection task. The CPAL is designed to assess working memory and proceeds in two stages. In the first stage, the subject must learn the locations of different pictures that are presented on screen. As each picture to be learned is revealed, the subject must tap each location and remember where the picture was located. In the second part of the task the same pictures are presented in the centre of the screen, and the subject must tap on the peripheral location where that picture previously appeared. The participant receives feedback on the accuracy of each response allowing them to update their selection until the correct location has been identified (the one-back and detection tasks have been described elsewhere, see Fredrickson et al) [21]. All tests were computerised and were administered by a research nurse or research psychologist in a sound-attenuated, dimly lit room. Participants were tested while seated in an armchair ∼65 cm from the computer screen. Time-of-day was held constant across all testing sessions for each subject. The order of test presentation within each session was also held constant. The Cogstate battery was always administered half way through the EEG battery. Participants were asked to abstain from caffeine and tobacco during the one-hour period prior to testing.

#### EEG acquisition

Continuous EEG was acquired through the ActiveTwo Biosemi electrode system from 64 scalp electrodes, digitized at 512 Hz. Vertical eye movements were recorded with two vertical electrooculogram (EOG) electrodes placed below the left and right eye, while electrodes at the outer canthus of each eye recorded horizontal movements. EEG data pre-processing and analysis were conducted using BESA Version 5.2 (Brain Electric Source Analysis) software. Blinks and eye movements were corrected using an algorithm developed by Berg and Scherg [70]. All electrode channels were average referenced offline and any segments with an amplitude deflection of greater than 90 mV were rejected in order to exclude excessive EOG or other noise transients.

#### Data analysis

For analysis and display purposes, data were average referenced and filtered with a low-pass 0-phase shift 48-dB, 40-Hz filter and a high-pass 0-phase shift 6-dB, 0.3-Hz. filter. Eyes-closed and eyes-open resting EEG band power was calculated using the discrete Fourier transform. Absolute power values are sensitive to confounds of head volume conduction and so to counteract this problem the use of relative power measures has been recommended [71], [72], [73], [74]. Relative power measures were also calculated by dividing the absolute power within the band by absolute power for the range of interest (1–30 Hz). Relative power was calculated separately for the Delta (1.0–3.5 Hz), Theta (4–7.5 Hz), Alpha 1 (8–10.5 Hz), Alpha 2 (10.5–13 Hz) and Beta (14–30 Hz) bands. Electrodes were grouped into separate frontal (F1, Fz, F2), central (C1, Cz, C2) and parietal (P1, Pz, P2) clusters.

#### Statistical analysis

Any missing values were replaced using the last observation carried forward method. Missing values occurred for two participants: in one case because the participant withdrew from the study before the final follow-up session and in the other because of excessively noisy data (<50% data yield) at the two week follow-up during the eyes open resting EEG tasks. Treatment effects on each of the cognitive and EEG variables were assessed using a repeated-measures ANCOVA. Factors included for the cognitive measures were Treatment (Donepezil vs Placebo) and Time (6-hours, 2 weeks, 4 weeks). Factors of Treatment, Time and Region (Frontal, Central, Parietal) were included for the EEG analyses. Baseline performance/band power was included as a covariate in each case as were age, MMSE score and estimated IQ. In cases where the Treatment effect reached significance a separate ANCOVA was conducted to explore acute drug effects at the 6-hour time point, with the same covariates as above. A Pearson correlation was run to assess the relationship between CPAL performance and resting EEG power measures which showed significant treatment effects. Individual performance/power measures were z transformed prior to the correlation analysis.

### Experiment 2: Double blind, crossover design, placebo-controlled trial of single dose (5 mg) donepezil administration using cognitive and simultaneous EEG/fMRI assessment

#### Participants

A total of twenty six healthy older participants were recruited to the study from the Dublin area. Eleven failed the initial screening and did not participate further in the experiment (same criteria as above – see Experiment 1, participants). One participant had corrupted EEG data and was excluded from the analysis. A final sample of fourteen participants (10 female) aged 55 to 76 (mean 67.93, SD = 7) were used for this study.

#### Screening

This study was a double-blind comparison of donepezil versus placebo within the same subjects across three separate testing sessions (Baseline (no dosage), Session 1 (donepezil or placebo), and Session 2 (alternative to session 1)). All participants completed the same medical and neuropsychological screening as in experiment 1.

On completion of screening, a randomisation procedure was used by the hospital pharmacy staff to allocate participants to receive either donepezil first or placebo first. All participants received both donepezil and placebo after completing both sessions. The drug and placebo capsules were over-encapsulated in order to make them indistinguishable. Subjects, test administrators and all medical staff were blinded to the treatment subjects received. Administration of the first dose took place in the hospital between 9:30 and 10 am. Subjects then returned on the same day at 3 pm for cognitive testing and EEG-fMRI (entered the scanner at 4 pm). Keeping scan time consistent helps to ensure a more morphologically stable BOLD response across sessions [75]. On completion of the study a full medical screening was repeated.

#### EEG and Cognitive Testing

Each testing session was identical and lasted 2 hours with approximately 1 hour of testing in the MRI scanner. Cognitive tests were administered outside the MRI scanner before EEG-fMRI tests. Only the CPAL was administered as this was the only cognitive task to show significant treatment effects in experiment 1. Participants then waited whilst the EEG cap was applied and electrode impedances reduced (below 10 kΩ, details in EEG-fMRI data acquisition below). The subject then entered the MRI scanner and performed an alternating eyes-open eyes-closed resting state, and three stimulus oddball task. The results of the three stimulus oddball will not be reported in this manuscript. The resting state task, inspired by Ben-simon et al., [22], had participants keep their eyes open for 30 s (during this time the screen said “OPEN” in large font). After 30 s the screen said “CLOSED” and participants closed their eyes. Participants then received a tap on the leg 30 s later to open their eyes again (once again the screen said “OPEN”). Visual instructions were used because piloting found that older participants often could not hear an auditory tone over the noise of the MRI scanner. This approach ensured that participants opened and closed their eyes as instructed. This resting state paradigm has the additional advantage of investigating two types of Alpha; Alpha reactivity and tonic Alpha, which have been shown to correspond to separate neural networks [22].

#### EEG/fMRI Apparatus

Subjects lay supine in an MRI scanner with the thumb of the right hand positioned on a two-button MRI-compatible response box. Stimuli were projected onto a screen behind the subject and viewed in a mirror positioned above the subjects face. Presentation software (Neurobehavioral Systems, Inc., USA) was used for stimulus presentation both inside and outside the scanner. A separate laptop running Brain Recorder v1.04 (BrainProducts, Munich, Germany) was used to record the EEG data at a frequency of 5 KHz (band-pass filtering from 0.016 to 250 Hz) along with event timings, response timings, and TTL pulses from the MRI scanner at the onset of each volume acquisition. These TTL pulses were also used to drive the visual stimuli in Presentation. The EEG clock was synchronised to the MRI scanner clock using the Brain products Sync-box [76]. Event timings and reaction times were calculated off-line using event timings acquired by Brain Recorder at this higher sampling frequency.

#### EEG/fMRI data acquisition

EEG recordings were acquired with a 32-channel MR-compatible BrainAmp system (Brainproducts, Munich, Germany). Thirty-one EEG electrodes were placed on the scalp not including the reference electrode positioned in correspondence to the FCz electrode (between electrodes Fz and Cz) and the ground electrode placed in correspondence to AFz (directly in front of Fz). One hangdown electrode was used to acquire the electrocardiogram (ECG). The impedance of all electrodes was maintained below 10 kΩ.

On the baseline sessions additional structural images were acquired. These included high-resolution T1-weighted anatomic MPRAGE image (FOV = 256*256*160 mm, thickness = 1 mm, voxel size  = 1 mm*1 mm*1 mm), T2-weighted FLAIR (FOV  = 230*143*184 mm, thickness = 4 mm (slice gap 1 mm), voxel = 0.45*0.45*6 mm), and T2-weighted spin echo (FOV = 230*143*201 mm, thickness = 4 mm (slice gap 1 mm), voxel = 0.45*0.45*6 mm). On every MRI session field maps were first acquired (two phase and magnitude images (TE_1_ = 1.46 ms, TE_2_ = 7 ms)). This was directly followed by the resting state EEG/MRI. The field of view covered the whole brain, 224*224 mm (64*64 voxels), 34 axial slices were acquired (0.05 mm slice gap) with a voxel size of 3.5 mm*3.5 mm*4 mm; TR = 2 s, TE = 32 ms, flip angle = 78°. This was a sparse-sampling sequence with the slices compressed to the first 1700 ms of the TR, leaving 300 ms uncontaminated by the MR gradient artefact. This was done to improve EEG data cleanup [77]. Each EPI session lasted 7 minutes (210 volumes). All MRI data was collected on a Philips 3T Achieva MRI Scanner (Centre for Advanced Medical Imaging (CAMI), St James's Hospital, Dublin).

#### EEG/fMRI pre-processing

EEG data was pre-processed using Analyser (BrainProducts, Munich, Germany) on an Intel Quad Core PC with 4GB of RAM, running Windows Vista SP2. Before pre-processing the data, the first TTL pulse of each EPI session was removed as it occurred at a different point in the gradient artefact template. After this, gradient correction was applied using the Average Artefact Subtraction (AAS) method [14], and a moving average of 25 windows. EEG data was then downsampled to 500 Hz and filtered between 0.53–30 Hz using IIR filters with an additional 50 Hz Notch filter. R-peaks were then detected in the ECG channel and removed using the AAS method [15], and a moving average of 11 artefacts. For both the gradient correction and balistocardiogram (BCG) correction multiple average window lengths were tried and these two were found to be the best based on decreased power at contaminated frequencies. Independent Components Analysis (ICA) was then run on the whole data set to remove artefacts including; blinks, eye movements, EMG and residual BCG artefacts [78]. The continuous data was then epoched based on TR markers (0–2000 ms) and artifactual epochs marked but not removed. An epoch was considered artifactual if the amplitude was ±80 µV, the voltage step was greater than 26 µV/ms, or activity lower than 0.5 µV. A discrete Fast Fourier Transform (FFT) was then run on each epoch, the results of which were then averaged.

Scans were pre-processed using SPM8 (www.fil.ion.ucl.ac.uk/spm) on an Intel Quad Core PC with 4GB of RAM, running Fedora 12 and Matlab 2009a (MathWorks Inc). Before pre-processing EPI data quality tests were run using the criterion defined in Iannetti et al., [79]. These include assessing the mean and standard deviation of the signal timecourse, image signal to noise, standard deviation of the single voxel signal timecourse, and visual inspection for ghost artefact and signal dropout. All EPI data collected passed these tests. Images were then realigned and unwarped using field maps to correct for motion artefacts, susceptibility artefacts and motion-by-susceptibility interactions [80], [81]. Images were subsequently normalized to the ICBM EPI template using the unified segmentation approach [82]. Lastly, a Gaussian kernel of 8 mm was applied to spatially smooth the image in order to conform to the Gaussian assumptions of a GLM as implemented in SPM8 [83], [84].

#### Statistical analysis

For analysis of both the CPAL and EEG frequency bands, a repeated measures ANOVA was conducted covarying for session (drug first or placebo first) and age. In the CPAL analysis baseline scores were also used as an additional covariate. For EEG frequency band differences a three way ANOVA was first run using factors of Treatment (Drug, Placebo), Frequency (Delta, Theta, Alpha 1, Alpha 2, Beta), and Location(Frontal (F3, F4, Fz), Central (C3, C4, Cz), Parietal (P3, P4, Pz), Occipital (O1, O2, Oz), Left Temporal (TP9, T7, P7), Right Temporal (TP10, T8, P8)). These were subsequently broken down into two way ANOVAs looking at each frequency band individually.

#### EEG/fMRI analysis

EEG power time courses were extracted using the same frequency boundaries as experiment 1 (Delta: 1.5–3.5 Hz; Theta: 4–7.5 Hz; Alpha 1: 8–11.5 Hz; Alpha 2: 12–13.5 Hz; Beta: 14–30 Hz). These timecourses were then downsampled to the TR sampling rate (0.5 Hz) and convolved with the informed basis set [85], [86]. Epochs previously marked as bad were replaced by the average of the two preceding and two proceeding data points. A general linear model (GLM) was constructed for each EEG-fMRI session consisting of the powertime course of each frequency band, convolved with three functions of the informed basis set (canonical hrf, temporal derivative, and dispersion derivative). Head movement parameters were also included. In order to assess drug related changes in tonic EEG (unaffected by eyes open vs eyes closed) a separate GLM was run which included all of the above regressors, plus an additional condition describing the changes between eyes open and eyes closed (this condition was modelled as three regressors due to the convolution with the informed basis set).

A second level random effects ANOVA was run using three factors; Treatment (donepezil or placebo), Time (session 1 or session 2); and Basis function (Canonical hrf, Temporal, and Dispersion derivative). Age was included as a co-variate given the drug*age interactions seen in the CPAL and the separate EEG analyses. Consistent with the literature [22], [62], [87], [88], [89], [90], [91], [92], results were thresholded at p<0.001 uncorrected, with an extent threshold of 10 unless otherwise specified. Anatomical localisation was performed using the anatomy toolbox [93]. Frequency response functions shown in figures 2, 3, 4 were calculated by multiplying the informed basis set with its respective Beta value from the peak of a cluster of interest.

## Supporting Information

Figure S1 **EEG drug effects for eyes open and eyes closed conditions.** a) Delta eyes closed drug effect. b) Alpha1 eyes closed drug effect. c) Delta eyes closed drug by age interaction. d) Alpha1 eyes closed drug by session interaction. e) Alpha2 eyes open drug by region interaction. An asterisk next to the region name indicate a significant difference (p<0.05). f) Alpha2 eyes open drug by region by session interaction. Circles highlight the driving force of the interaction. g) Alpha2 eyes open drug by region by session interaction. Drug placebo differences are plotted. Central and right temporal differences are highlighted as they drive the drug by region by session interaction. In plots a–e red represents relative EEG power on donepezil, blue represents placebo.
(DOCX)

Table S1 **CPAL performance for experiment 1 and experiment 2.** Values are percent accuracy with values in brackets showing the standard error.
(DOCX)

Results S1 **Experiment 2 EEG results for drug effects on tonic EEG power and EEG reactivity.**
(DOCX)
