# Supplementary material for: Donepezil Impairs Memory in Healthy Older Subjects: Behavioural, EEG and Simultaneous EEG/fMRI Biomarkers
Source: PLoS One. 2011 Sep 8;6(9):e24126. doi: 10.1371/journal.pone.0024126 (PMC3169575; doi:10.1371/journal.pone.0024126)
Supplement: Figure S1 — EEG drug effects for eyes open and eyes closed conditions. a) Delta eyes closed drug effect. b) Alpha1 eyes closed drug effect. c) Delta eyes closed drug by age interaction. d) Alpha1 eyes closed drug by session interaction. e) Alpha2 eyes open drug by region interaction. An asterisk next to the region name indicate a significant difference (p<0.05). f) Alpha2 eyes open drug by region by session interaction. Circles highlight the driving force of the interaction. g) Alpha2 eyes open drug by region by session interaction. Drug placebo differences are plotted. Central and right temporal differences are highlighted as they drive the drug by region by session interaction. In plots a–e red represents relative EEG power on donepezil, blue represents placebo. (DOCX) [file pone.0024126.s001.docx]

**Figure S1: EEG drug effects for eyes open and eyes closed conditions.** a) Delta eyes closed drug effect. b) Alpha1 eyes closed drug effect. c) Delta eyes closed drug by age interaction. d) Alpha1 eyes closed drug by session interaction. e) Alpha2 eyes open drug by region interaction. An asterisk next to the region name indicate a significant difference (p<0.05). f) Alpha2 eyes open drug by region by session interaction. Circles highlight the driving force of the interaction. g) Alpha2 eyes open drug by region by session interaction. Drug placebo differences are plotted. Central and right temporal differences are highlighted as they drive the drug by region by session interaction. In plots a-e red represents relative EEG power on donepezil, blue represents placebo.

d)

c)

b)

a)

e)

*

g)

f)
