# Supplementary material for: Donepezil Impairs Memory in Healthy Older Subjects: Behavioural, EEG and Simultaneous EEG/fMRI Biomarkers
Source: PLoS One. 2011 Sep 8;6(9):e24126. doi: 10.1371/journal.pone.0024126 (PMC3169575; doi:10.1371/journal.pone.0024126)
Supplement: Table S1 — CPAL performance for experiment 1 and experiment 2. Values are percent accuracy with values in brackets showing the standard error. (DOCX) [file pone.0024126.s002.docx]

**Table S1:** CPAL performance for experiment 1 and experiment 2. Values are percent accuracy with values in brackets showing the standard error.

Experiment 1:

|  | **Baseline** | **6 hours** | **2 weeks** | **4 weeks** |
| --- | --- | --- | --- | --- |
| **Drug** | 0.423 (0.04) | 0.428 (0.05) | 0.419 (0.04) | 0.447 (0.04) |
| **Placebo** | 0.516 (0.05) | 0.631 (0.08) | 0.683 (0.07) | 0.648 (0.06) |

Experiment 2:

| **Baseline** | **Drug** | **Placebo** |
| --- | --- | --- |
| 0.680 (0.03) | 0.715 (0.04) | 0.722 (0.05) |
