# Supplementary material for: Donepezil Impairs Memory in Healthy Older Subjects: Behavioural, EEG and Simultaneous EEG/fMRI Biomarkers
Source: PLoS One. 2011 Sep 8;6(9):e24126. doi: 10.1371/journal.pone.0024126 (PMC3169575; doi:10.1371/journal.pone.0024126)
Supplement: Results S1 — Experiment 2 EEG results for drug effects on tonic EEG power and EEG reactivity. (DOCX) [file pone.0024126.s003.docx]

**Results S1: Experiment 2 EEG results for drug effects on tonic EEG power and EEG reactivity.**

Experiment 2 used a different resting task to experiment 1 where the participant alternated between eyes-open and eyes-closed conditions every 30s. This allowed us to model two types of EEG oscillatory markers within each frequency band; EEG reactivity (EEG power that changed between eyes-open and eyes-closed conditions) and tonic EEG power (EEG power unaffected by the transition between eyes-open and eyes-closed conditions). Two way ANOVAs were then run on each EEG frequency band separately.

For the reactivity analysis, resting Theta, Alpha1, and Alpha 2 showed significant effects. Theta EEG power showed significantly reduced reactivity on donepezil (F(1,11) = 5.59, p<0.05, η^2^=0.34). There was also a treatment by age interaction showing reduced reactivity for participants in their 60’s on donepezil and increased reactivity for participants in their 70’s on donepezil, (F(1,11) = 5.22, p<0.05, η^2^=0.32). Alpha 1 EEG reactivity was significantly reduced on donepezil (F(1,11) = 14.46, p<0.005, η^2^=0.57). There were also a number of treatment by drug interactions including treatment by age (F(1,11) = 6.59, p<0.05, η^2^=0.37; donepezil had a stronger negative effect (reducing reactivity) for participants in their 50’s and 60’s), treatment by region (F(5,55) = 3.13, p<0.05, η^2^=0.22; driven by significantly reduced reactivity on donepezil in Occipital regions) and treatment by session (F(1,11) = 13.47, p<0.005, η^2^=0.55; drug effects disappeared if donepezil was administered first). Finally, Alpha 2 EEG reactivity also showed a significant treatment by session interaction (F(1,11) = 5.72, p<0.05, η^2^=0.34; Alpha2 EEG reactivity was lost in the placebo condition if donepezil was administered first).

Tonic resting EEG showed significant treatment effects for Delta, Alpha2 and Beta. Tonic Delta EEG power significantly increased on donepezil (F(1,11) = 6.55, p<0.05, η^2^=0.37). There was also a treatment by age interaction showing that tonic Delta EEG power was reduced for participants in their 70’s (F(1,11) = 5.19, p<0.05, η^2^=0.32). Tonic Alpha2 resting EEG showed treatment by region (F(5,55) = 3.12, p<0.05, η^2^=0.22; driven by significantly reduced tonic Alpha2 EEG power in Frontal regions), and a treatment by region by age interaction (F(5,55) = 4.05, p<0.005, η^2^=0.27) driven by drug by age interactions in the right temporal region. Tonic Beta EEG power also showed a significant treatment by age interaction due to decreasing tonic Beta EEG power with age on placebo and increasing tonic Beta EEG power with age on donepezil (F(1,11) = 5.88, p<0.05, η^2^=0.35).
